# From mice to clinical relevance: humanizing neuroscience with human-based model systems

**DOI:** 10.3389/fncel.2026.1776439

**Published:** 2026-04-02

**Authors:** Aniella Vanessa Bak, Karen M. J. van Loo, Deborah Kronenberg-Versteeg, Henner Koch

**Affiliations:** 1 Department of Epileptology, Neurology, RWTH University Hospital Aachen, Aachen, Germany; 2 Department of Neurosurgery, RWTH University Hospital Aachen, Aachen, Germany; 3 Department of Cellular Neurology, Hertie Institute for Clinical Brain Research, University of Tübingen, Tübingen, Germany; 4 German Center for Neurodegenerative Diseases (DZNE), Tübingen, Germany

**Keywords:** brain-on-a-chip, human brain tissue, human-based models, IPSC, organoid, species differences, translational neuroscience

## Abstract

Preclinical research in neuroscience has traditionally relied on animal models to investigate disease mechanisms and develop new therapeutic strategies. While these models are valuable to gain mechanistic insights, their translational power remains limited due to interspecific differences and, hence, frequent failures in clinical translation. The uniqueness of the human brain calls for alternative approaches in neuroscientific research that more faithfully capture human physiology and pathology. In recent years, a variety of human-based model systems have emerged, ranging from dissociated neuronal cultures and stem cell-derived platforms, including organoids, to micro-engineered devices and human brain slice approaches. Each model offers distinct advantages and limitations in recapitulating neural circuits, disease mechanisms, and therapeutic responses. In this review, we critically discuss the merits and drawbacks of animal models, outline the historic development and current applications of human-based systems, and highlight their potential to complement or replace animal-based models. We further explore current challenges in human brain research, including human variability, technical challenges, as well as ethical considerations and regulatory hurdles. Together, these advances represent a shift toward more predictive, human, and ethically responsible neuroscientific research that could aid in decreasing the translational gap.

## 1 Introduction

The human brain is one of the most complex and unique organs, presenting significant challenges for preclinical research due to its limited accessibility. Hence, neuroscience depends on model systems to investigate cellular, circuit, and systems-level mechanisms in physiology and pathology under controlled conditions. These model systems serve as cornerstones in neuroscientific research to observe the status quo, identify disease mechanisms, and develop new therapeutic approaches for neurological diseases. A multitude of model systems is available for this cause, from simplistic cell cultures to brain tissue samples and complete *in vivo* animal models, depending on the research question and biological complexity required.

While traditional animal-based models have made invaluable contributions to neuroscience, they face growing criticism regarding their validity and relevance. The human brain is unique and complex in its features, making it difficult to represent human neurobiology accurately in animal-based model systems. In addition, animal welfare concerns surrounding the use of animals for scientific research have grown during recent years. Consequently, an urgent need has emerged for more human-relevant experimental platforms in order to complement or replace certain animal models traditionally used in neuroscience.

In this review, we use the term “translation” to refer to a multi-dimensional process including mechanistic insight, human specificity, and predictive value for therapeutic development, rather than a strictly linear progression toward clinical application. Human-specific model systems function as pre-translational filters by enabling human-relevant investigation of these parameters, thereby strengthening preclinical decision-making and potentially reducing the translational gap in neuroscience. Importantly, increased biological complexity or technological sophistication does not directly correspond to reduced translational distance to clinical decision-making. Instead, the suitability of a given model system is inherently dependent on the biological context and the specific research question addressed. In the following sections, we therefore discuss the position, advantages and disadvantages of animal model systems in neuroscientific research, followed by the major human-based or “humanized” model systems in detail, highlighting their respective strengths, limitations, and optimal areas of application to facilitate informed model selection for translational neuroscience research.

## 2 Animal models in preclinical research

In neuroscientific preclinical research, a multitude of animal models are used, the species ranging from non-human primates to rodents and lower animals, such as invertebrates. Each animal model comes with advantages and limitations, and choosing the right model system depends on the specific research question. *C. elegans* is used to study basic disease mechanisms (Fanning et al., 2019) and simple neural circuit dynamics (Zullo et al., 2019). *Drosophila melanogaster* serves as a powerful model for investigating genetic and molecular mechanisms underlying disease, such as genetic epileptic encephalopathies (Karge et al., 2025), and can also function as a high-throughput platform for screening potential therapeutics (Fischer et al., 2024). Zebrafish are valuable for studies of neural development (Lange et al., 2024) and for validating disease processes, such as multiple sclerosis (Pahlevan Kakhki et al., 2024), due to their translucidity and rapid development. Songbirds, such as zebra finches, are used to study learning and memory (Daou and Margoliash, 2020), providing insight into neuroplasticity (Lu et al., 2023). To investigate elevated social or cognitive behaviors like addiction (Ding and Ko, 2021), as well as complex multi-factorial diseases such as autism (Watanabe et al., 2021), non-human primates can be employed as well. One of the main pillars of neuroscientific research, however, are rodents, which are widely used and invaluable in biomedical research. Despite the differences in size and complexity, the human and mouse brain share their basic anatomical organization and neurotransmitter systems (Krubitzer, 2007). Major brain cell type classes and subclasses are highly conserved between humans and mice, albeit with some differences in proportions, gene expression, morphology, and laminar distribution (Hodge et al., 2019). Furthermore, many fundamental processes underlying brain development, function, and behavior are evolutionarily conserved between humans and mice (Semple et al., 2013). Basic properties of functional connections, plasticity, and excitation/inhibition are also largely conserved (Mansvelder et al., 2019; Kim et al., 2023). Additionally, rodents are small animals that can be easily bred; they exhibit short generation times and produce large litters, facilitating studies across multiple generations in a relatively short time frame, which is especially beneficial for studying genetic mutations or defects (Yan et al., 2021). Genetically modified strains are readily available to model human diseases of the central nervous system (CNS) and to investigate pathogenesis, underlying mechanisms, and potential treatments (Brooks et al., 2021; Marshall et al., 2021; Gustavsson et al., 2023). Generally, *in vivo* mouse models enable the study of complex brain-wide mechanisms, such as brain area connectivity, neuronal subpopulation analysis, or modeling of complex diseases such as developmental and epileptic encephalopathies (Kramvis et al., 2020; Kramer et al., 2021; Musall et al., 2023). They also allow investigation of cross-talk between multiple organ systems, which is believed to play a crucial role in many diseases.

Despite the similarities mentioned above and the convenience of using rodents for neuroscientific research, the human brain is exceptionally unique (van Loo et al., 2025). It is structurally the most complex organ known and therefore presents neuroscientists with enormous challenges. Access to live human brain tissue is rare and difficult to obtain for experimental purposes, necessitating the use of alternative model systems. Consequently, rodent models have long served as the method of choice in neuroscientific research. Nevertheless, due to significant anatomical, molecular, and functional differences between human and rodent brains, it is essential to critically evaluate the translational relevance of these models.

The human brain differs from that of the mouse in both size and complexity. One of its most distinctive features is its folded outer layer, the cerebral cortex, which governs higher cognitive functions such as thought, language, and abstract thinking (Akula et al., 2023). Additionally, the human brain exhibits a greater diversity of neuronal connections and cell types (Miller et al., 2019), e.g., the recently described rosehip cell (Boldog et al., 2018), which up to date, has not been reported in rodent brains. This diversity is also reflected in gene expression and regulation, with many genes associated with human-specific cognitive functions differing or remaining unexpressed in the mouse (Strand et al., 2007; Bakken et al., 2021). Moreover, human cortical pyramidal neurons display specific membrane properties and signal processing compared to rodents (Eyal et al., 2016). For example, a human-specific subset of L2/3 neurons has been described in the cortex as showing longer dendritic length and more substantial signal attenuation than in the mouse (Mohan et al., 2015), while human L5 neurons deviate from the allometric rules of conserved conductance per unit brain volume as seen in other mammals, exhibiting much lower voltage-gated potassium and hyperpolarization-activation cyclic nucleotide-gated (HCN) conductances (Beaulieu-Laroche et al., 2021).

For translational research, these species-specific differences are critical. The complexity and heterogeneity of human multifactorial diseases often cannot be fully captured by mouse models (Leenaars et al., 2019; Azkona and Sanchez-Pernaute, 2022). Moreover, interspecies variation in drug metabolism and receptor pharmacology leads to considerable discrepancies in drug responses (Uhl and Warner, 2015), which contribute to the strikingly high clinical trial failure rates of CNS-targeted drugs derived from animal studies (Hay, 2014).

In addition to translational limitations, animal welfare concerns have gained increasing attention in recent years. The factual benefit of using animals for experimental research in light of their welfare costs is subject to ongoing debate (Andersen and Winter, 2017; Kiani et al., 2022). Although complete elimination of animal models from research is unimaginable at this point, and strict rules concerning animal welfare and suffering are in place, many efforts have been made to improve *in vitro* approaches (Gan et al., 2022; Muralidharan, 2021). These efforts align with the principles of the 3Rs—replacement, reduction, and refinement—aiming to reduce animal numbers, refine experimental procedures, and ultimately replace *in vivo* approaches where possible (Díaz et al., 2021).

**Figure 1:**
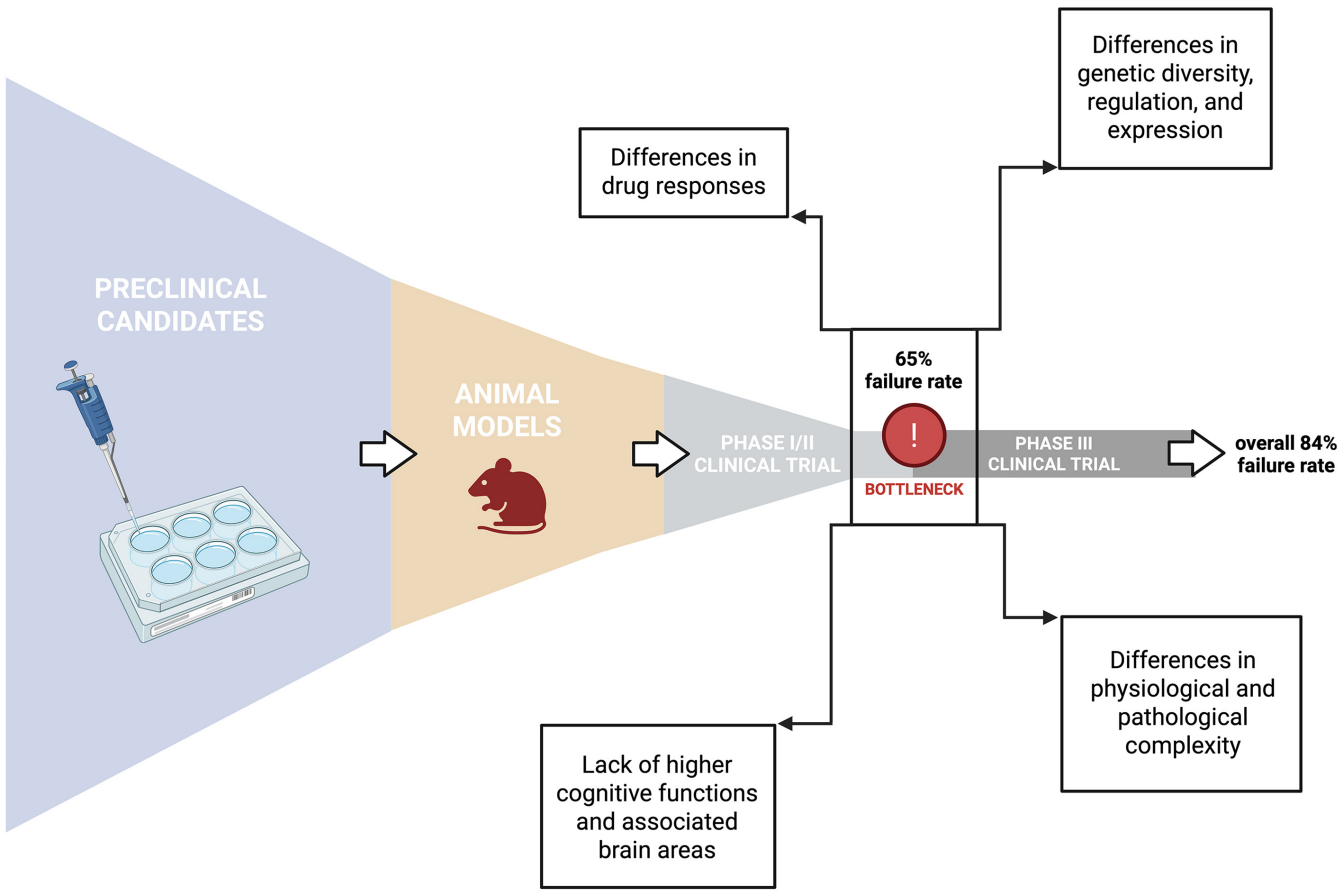
The translational gap in neuroscience. Despite the multitude of preclinical candidates for new CNS drugs and therapeutic interventions (379 phase I initiations from 1990 to 2012), and while only 5% fail in phase I clinical trial evaluation for human safety (361 phase II initiations), another staggering 60% fail in phase II, leaving only 35% of overall candidates (132 phase III initiations) to enter phase III clinical trials, where another 19% of the initial pool of candidates are discontinued. Most of these failures are attributed to a lack of efficacy in the human system (Kesselheim et al., 2015), due to severe translational hurdles during the transition from animal models to human patients.

That this transition toward human-relevant systems is both timely and necessary becomes evident when considering the limited translational success of traditional animal research in CNS research, considering that 84% of preclinical candidates fail to transition from phase I to phase III clinical trials (Kesselheim et al., 2015) (Figure 1). In conclusion, rodent models remain a valuable resource for gaining mechanistic insight into basic neurobiology and disease processes, and the only resource for behavioral readouts. Their short life cycle, genetic tractability, and experimental accessibility continue to make them powerful tools for hypothesis testing, mechanistic exploration, and methodological validation. However, findings derived from animal systems must ultimately be verified and extended in human-based or humanized models, which provide essential translational context and capture the unique complexity of the human brain.

The following chapters will explore a range of human-derived *ex vivo* and *in vitro* models currently employed in neuroscientific research, their evolution, along with their respective advantages and limitations. We highlight their emerging role in strengthening preclinical research by enabling human-specific mechanistic insights and validation of candidate interventions. By evaluating each model system in a question-driven context, we aim to guide readers in selecting the most suitable platform for their experimental objectives, thereby facilitating more informed and translationally relevant study design.

## 3 Humanizing neuroscience model systems

**Figure 2:**
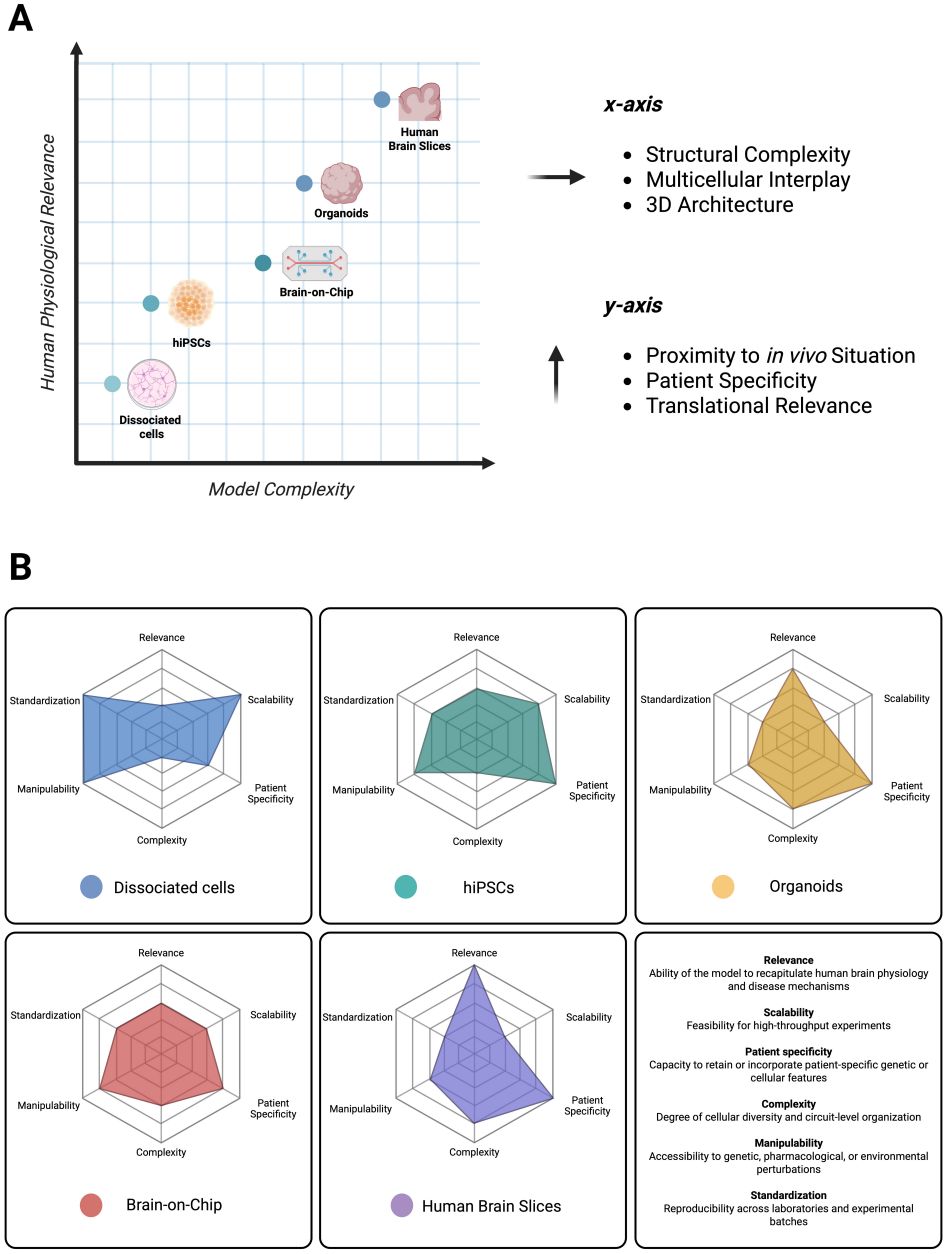
Trade-off between practical attributes and key strengths of human-based model systems. **(A)** Graph depicting model complexity (x-axis, including structural complexity, multicellular interplay, and 3D architecture) in relation to human physiological relevance (y-axis, including proximity to *in vivo* situation, patient specificity, and translational relevance). **(B)** Radar plots illustrating individual strengths of different model systems concerning relevance, scalability, patient specificity, complexity, manipulability, and standardization, based on the insights presented in this review. Color-coded for dissociated cells (blue), hiPSCs (green), organoids (yellow), brain-on-chip (red), and human brain slices (violet). Each parameter was scored on a semi-quantitative scale from 1 (very low) to 5 (very high). Scores reflect an expert-informed synthesis of published literature (Supplementary Table 1). Scores were assigned based on consensus reported in the literature, including methodological reviews, and reported use cases.

The use of human-based experimental systems has gained increasing importance in neuroscience. These models offer the opportunity to directly investigate human neuronal properties, disease mechanisms, and pharmacological responses within their native molecular and genetic context. Over the past decades, advances in tissue culturing techniques, stem cell biology, and bioengineering have enabled the establishment of diverse *in vitro* and *ex vivo* model systems that capture different levels of neural organization and complexity, from single cells to functional networks (Figure 2A), and therefore each provide a specific repertoire of strengths regarding relevance, scalability, patient specificity, complexity, manipulability, and standardization (Figure 2B). The following sections will outline the major types of human-derived model systems currently used in neuroscience, beginning with the most fundamental level: dissociated human neuronal cultures.

### 3.1 Dissociated cell cultures

#### 3.1.1 Historical development of dissociated systems, experimental strengths and limitations

Dissociated neuronal cultures represent one of the oldest and most widely used *in vitro* systems in neuroscience. They can be obtained either as commercially available, immortalized cell lines or can be created directly from primary tissue. Historically, primary dissociated neurons have been predominantly derived from rodent embryonic or early post-natal tissue, enabling robust protocols for cortical (Huettner and Baughman, 1986), hippocampal (Banker and Cowan, 1977), and cerebellar (Tabata et al., 2000) neurons. These cultures maintain essential phenotypic markers, develop elaborate dendritic and axonal arbors, and form functional, synaptically connected networks *in vitro* (Beaudoin et al., 2012). Because experimental parameters such as growth factors, extracellular matrix components, or pharmacological agents can be precisely controlled (Yang et al., 2014), dissociated rodent primary neurons provide an excellent reductionist platform for mechanistic studies. The relative homogeneity of neuronal populations further facilitates data interpretation when compared to the intact brain *in vivo* or isolated specimen thereof, which contain various cell types and therefore react in more complex patterns to any kind of manipulation. Additionally, they provide good optical accessibility for high-resolution live imaging of neuronal morphology, plasticity, and transport (Brown, 2003; Yamamoto et al., 2012; Verstraelen et al., 2018). As a result of these strengths, dissociated primary rodent neurons have been widely used to investigate neuronal development, also in the context of developmental and epileptic encephalopathies, aging and death, neurodegeneration, and neurotoxicity (Lesuisse and Martin, 2002; Chiappalone et al., 2006; Silva et al., 2006; Schonfeld-Dado and Segal, 2009; Liu et al., 2021).

Yet, fundamental limitations remain: dissociation disrupts the three-dimensional architecture and cellular diversity of the brain tissue, leading to complete loss of *in vivo* context. The formation of circuits is functional yet oversimplified and does not represent the elaborate network connectivity and dynamics found in intact brain tissue (Chow et al., 2022). Furthermore, as dissociated neurons often originate from embryonic or early post-natal tissue, they exhibit an immature phenotype and only mature over time in culture (Ichikawa et al., 1993) with spontaneous activity patterns that appear dependent on neuron density (Wagenaar et al., 2006; Cullen et al., 2010). As spontaneous electrical activity is coupled to neuronal maturation (Bergey et al., 1981), the culturing process severely influences neuronal development and can lead to immense variability between different cultivation approaches and protocols (Sünwoldt et al., 2017). These challenges are amplified when moving from rodent to human tissue. The sensitivity and limited lifespan of cells, the need for very strict culturing conditions and medium formulation, as well as batch-to-batch variation, introduce additional variability and cellular stress, as highlighted by recent overviews of primary brain cell isolation (Aggarwal et al., 2025).

Moreover, although the conceptual framework of dissociated cultures is shared across species, the feasibility of primary adult human neuronal cultures differs fundamentally from that of rodent neurons. The first successful attempts to isolate, dissociate, and culture human adult neurons were reported by Kim et al. (1979), who managed to culture human trigeminal and superior ganglia neurons for more than 2 months. Over subsequent decades, protocols originally developed for the culture of rodent neurons were further refined to improve the survival and differentiation of human neuronal cells, allowing for better synaptogenesis and maturation (Brewer et al., 2001). Continuous efforts have since aimed to further improve culturing conditions to promote mature neurophysiological properties, such as fast-spiking behavior and spontaneous synaptic activity (Park et al., 2020). In parallel, the introduction of single-cell transcriptomic analysis of primary human brain cells has opened completely new avenues for molecular characterization of these cultures, providing insights into cell identity, heterogeneity, and maturation states (Spaethling et al., 2017). Adult human neural cells obtained after traumatic brain injury can also be maintained in culture over several days with preserved morphology and cytoskeletal organization (Freire et al., 2023), underscoring the general feasibility of such preparations under optimized conditions. Comparable protocols have been developed for primary sensory neurons, for instance, the surgical extraction of human dorsal root ganglia from organ donors and their use for primary sensory neuron culture (Valtcheva et al., 2016).

Despite these methodological advances, primary adult human neuronal cultures remain rare. Their use is constrained by ethical and practical limitations to access viable adult human brain tissue, which is typically restricted to small surgical resections or post-mortem tissue. Once removed from their native environment, adult neurons, due to being post-mitotic, structurally fragile, and extremely dependent on synaptic and glial networks, rapidly deteriorate without elaborate protocols. Furthermore, each human sample is donor-specific and non-renewable, impacting standardization and reproducibility. Moreover, adult primary cultures lack developmental trajectories, making them unsuitable for modeling neurodevelopmental disorders, where human-specific developmental programs and circuit assembly are critical (Zhao and Bhattacharyya, 2018).

By contrast, human astrocytes and microglia are more robust in culture and therefore more commonly used as dissociated human systems. Enriched primary human microglia preparations, while retaining some key effector functions, including phagocytosis and responsiveness to inflammatory stimuli (Rustenhoven et al., 2016), however, demonstrate significant transcriptional changes in their microglial signatures within hours of being taken into culture (Gosselin et al., 2017). Nevertheless, phenotypic characterization has revealed that primary microglia display distinct inflammatory and anti-inflammatory profiles compared to immortalized microglial lines (Melief et al., 2016). Human astrocytes isolated and characterized from neurotrauma patients express appropriate astrocytic markers and can be used to study human-specific astroglial functions *in vitro* (Gradisnik et al., 2020). In parallel, immortalized microglial lines such as human HMO6, alongside primary microglial cultures, can, to some extent, provide experimentally valid models to investigate microglial activation and neuroinflammation, particularly in Alzheimer's disease (AD) (Stansley et al., 2012). Together, these studies highlight that while neuronal cultures are theoretically central to dissociated systems, in practice, human glial cultures often offer greater feasibility and robustness.

To fill the gap of human dissociated neuronal cultures, immortalized human neuronal cell lines are an important alternative. The best-known example is the SH-SY5Y line, a thrice-subcloned derivative of the SK-N-SH neuroblastoma cell line, originally established in 1970 from a metastatic bone marrow biopsy of a 4-year-old female with neuroblastoma (Biedler et al., 1973). Through various differentiation protocols, SH-SY5Y cells can acquire cholinergic (D'Aloia et al., 2024), dopaminergic (Presgraves et al., 2004), adrenergic (Khazeem et al., 2022), or glutamatergic (Martin et al., 2022) properties, offering a flexible and scalable human cell system for diverse experimental applications. Other neuron-like lines, such as the Lund human mesencephalic (LUHMES) line and the NT2/D1 line with their neuronal and astrocytic derivatives, further expand the available repertoire of human neuron- and glia-like cell models (Lotharius et al., 2002; Taylor et al., 2019; Balint et al., 2024). Reviews have summarized how such neuron-like cell lines and primary neuron models complement each other in research by combining feasibility with physiological relevance (Xicoy et al., 2017; Ghiasvand et al., 2024).

Immortalized cell lines offer several advantages compared to primary neuronal cultures. For example, they can be of human origin and infinitely passaged, unlike mature neurons in primary cultures, which do not undergo cell division, resulting in a limited cell number per preparation. Additionally, adeno-associated vectors are more efficiently transduced in immortalized cell lines (Halbert et al., 1995). Cell lines are generally well-characterized, exhibit low variability within the culture and between strains, and allow reproducible results, which makes them attractive for standardized assays, toxicological evaluation, and mechanistic screening (Carter and Shieh, 2015; Lopez-Suarez et al., 2022). However, using immortalized cell lines comes with notable drawbacks such as cross-contamination (Jiang and Wang, 2014), misidentification (Alston-Roberts et al., 2010), and genetic drift accompanied by loss of genetic and phenotypic identity (Ross et al., 2000). Specifically, the SH-SY5Y immortalized human neuronal cell line retains properties of neuronal precursors and lacks proper neurite and synapse formation (Gordon et al., 2013) unless exposed to an elaborate proliferation protocol (Dravid et al., 2021; Targett et al., 2024). In addition, differentiation route-dependent variability in key readouts, such as susceptibility to amyloid-beta toxicity, has been reported as a model-specific pitfall (Krishtal et al., 2019).

In conclusion, the historical development of dissociated neuronal cultures from rodent embryonic preparations to more rare primary adult human neurons and a diverse spectrum of immortalized human cell lines has yielded a versatile set of *in vitro* tools. Primary rodent cultures offer high experimental control and reproducibility, but lack human specificity, while primary adult human neurons are conceptually attractive but constrained in tissue access, fragility, and donor variability. Immortalized human lines provide scalability and standardization, but at the expense of full physiological relevance. These systems collectively reveal fundamental principles of neuronal and glial function, yet are oversimplified, lack 3D cytoarchitecture, and multicellular complexity. Nevertheless, dissociated systems have become indispensable for mechanistic studies in physiology and pathophysiology, as well as for high-throughput screening methods.

#### 3.1.2 Dissociated systems in human neuroscience and disease modeling

Despite the loss of *in vivo* context and their simplistic organization, dissociated cultures remain widely used due to their accessibility, experimental flexibility, and reproducibility (Lopez-Suarez et al., 2022; Ghiasvand et al., 2024). Both primary dissociated neurons and immortalized human neuronal cells have been used extensively to study the cellular basis of neurological disorders. While primary neurons provide physiologically and genetically relevant and well-differentiated systems (Ray et al., 2014), immortalized cell lines offer standardization, scalability, and reproducibility, making both approaches complementary in neuroscientific disease research.

##### 3.1.2.1 Neurodegenerative disorders

Dissociated neuronal cultures have been fundamental in the investigation of pathological mechanisms underlying neurodegenerative diseases. In Parkinson's disease (PD), the SH-SY5Y immortalized cell line, capable of dopaminergic or cholinergic differentiation (Presgraves et al., 2004; Alrashidi et al., 2021), remains widely used to model oxidative stress, mitochondrial dysfunction, and proteasome inhibition, as well as to evaluate neuroprotective strategies (Table 1, a, b). The LUHMES cell line, a conditionally immortalized human mesencephalic line, provides a more mature dopaminergic model suitable for investigating alpha-synuclein aggregation, oxidative stress, and genetic PD mechanisms such as LRRK2 mutation G2019S (Lotharius et al., 2002; Calamini et al., 2021). LUHMES cells have also been adapted into screening platforms targeting mitochondrial dysfunction (Leah et al., 2021) and into 3D dopaminergic constructs for long-term neurotoxicity testing (Smirnova et al., 2016). Comparative studies additionally indicate that LUHMES cells exhibit greater phenotypic stability and responsiveness to PD-relevant stimuli than SH-SY5Y (Keighron et al., 2024).

**Table 1:** Application examples for section 3.1 on dissociated cell culture systems.

Ref.	Model system	Cell source/line	Application/ disease	Study focus	Key findings	References	Article type
a	Immortalized neuronal line	SH-SY5Y	Parkinson's disease	Neuroprotection against oxidative stress, mitochondrial dysfunction, lysosome and proteasome inhibition	Classical compounds (caffeine, creatine, nicotine, coenzyme Q10, deprenyl) mitigate neuronal damage	Yong-Kee et al., 2011	Original article
b	Immortalized neuronal line	SH-SY5Y	Parkinson's disease	Testing neuroprotective effects of CDK inhibitor in rotenone-induced PD model	CDK inhibitors reduce ROS, apoptosis and caspase activation	Yerramsetty et al., 2025	Original article
c	Immortalized neuronal line	SH-SY5Y	Alzheimer's disease	Therapeutic intervention and evaluation of neuroprotective effects of different compounds in cell-based AD models	Neuroprotective potential of different compounds in AD, alleviating oxidative stress, apoptosis, mitochondrial dysfunction, and inflammation	Pang et al., 2023; Pieńkowska et al., 2023; Nopparat et al., 2023; Zhang G. H. et al., 2023; Huang et al., 2014; Md et al., 2018; Gandbhir and Sundaram, 2020	Original article
d	Immortalized neuronal line	SH-SY5Y	Alzheimer's disease	Modulation of miR-6076 and BCL6 in an AD cell model	Downregulation of miR-6076 reduces p-Tau and apoptosis	Lin et al., 2023	Original article
e	Immortalized neuronal line	SH-SY5Y	Alzheimer's disease	Study of APP V225A mutation effects on AD hallmarks	APP V225A promotes AD pathology, identification of key therapeutic target	Chen et al., 2025	Original article
f	Immortalized neuronal line	SH-SY5Y	Neurodevelopment/ Toxicology	Neurodevelopmental effects of antiepileptic drugs	Carbamazepine and Lamotrigine show toxic effect; potential impact on fetal development by valproic acid	Kakumoto et al., 2021	Original article
g	Immortalized neuronal line	SH-SY5Y	Toxicology	Neuroprotective effects of pharmacological agents against chemically induced neuronal injury	Protective effects of pantoprazole, valproic acid, and levetiracetam against seizure- or chemical-induced neuronal damage	Taskiran et al., 2021; Ahlatci et al., 2022; Çiltaş et al., 2022	Original article
h	Immortalized neuronal line	HBEC-5i	Blood-brain-barrier function and drug delivery	ABC transporter activity and validation with pharmacological substrates	Functional transporter activity, enhanced by astrocyte-conditioned medium, model validated with caffeine, rivaroxaban, methotrexate	Puech et al., 2018	Original article
i	Immortalized cell line	PC12, SH-SY5Y, BV2, HA, HBMEC	Neuroinflammation	2D and 3D co-culture systems, modeling healthy vs. Inflamed BBB	Overview of *in vitro* tools for anti-inflammatory and neurological research	Peng et al., 2021	Review
j	Primary human cells	astrocytes, pericytes, HBMEC, neurons	Blood-Brain-Barrier	Creation of a novel transwell BBB model using primary human cells	Four cell model including neurons is more representative of interactions at the neurovascular unit than two- and three cell models	Stone et al., 2019	Original article
k	Immortalized cell line	HBMEC/ci18, HASTR/ci35, HBPC/ci37	Blood-Brain-Barrier	Development of a human immortalized cell-based BBB tri-culture model	Model exhibits essential BBB functionality, availability of immortalized lines enhanced reproducibility and standardization	Ito et al., 2019	Original article

In AD research, SH-SY5Y-based models are extensively used to study amyloid-β and tau pathology and to investigate neuroprotective and disease-modifying mechanisms, such as the A673T mutation in the amyloid precursor protein (Guyon et al., 2020). These model systems have also been widely used to support mechanistic studies of oxidative stress, inflammatory signaling, and pathway-specific modulation of tau phosphorylation and amyloidogenesis, as well as to test therapeutic interventions (Table 1, c–e). Additionally, proteomic profiling has revealed a neuroprotective effect of melatonin on amyloid-beta-induced mitochondrial impairment, intracellular peptide accumulation, and tau hyperphosphorylation (Panmanee et al., 2025), while secretomic analysis documented amyloid-beta-induced changes in AD-related proteins (Roytrakul et al., 2024). Notably, the SH-SY5Y differentiation route seems to influence responses to amyloid-beta, representing an important limitation of the model (Krishtal et al., 2019).

More complex human-derived systems extend dissociated AD modeling to three dimensions. Human progenitor-derived or human neural stem cell line “ReN”-derived 3D cultures carrying familial AD mutations recapitulate amyloid-beta ratio-dependent tau pathology and permit systematic analysis of plaque-tangle interactions (Kim et al., 2015; Kwak et al., 2020). Tau seeding approaches using AD patient-derived tau filaments demonstrated templated pathological tau aggregation in SH-SY5Y cells, supported by cryo-EM characterization of disease-specific filament structures (Tarutani et al., 2023). Extensions to LUHMES-based tauopathy models (Moffett and Parmar, 2024), and cortical-like SH-SY5Y derivatives (Devyatov et al., 2025) further broaden the applicability of dissociated systems for studying tau aggregation dynamics.

##### 3.1.2.2 Mechanistic studies, toxicology, and drug screening

Beyond disease-specific mechanisms, dissociated neuron-like cells have also been extensively used to assess developmental neurotoxicity, neuroprotection, and drug safety (Lopez-Suarez et al., 2022; Monteiro et al., 2024). SH-SY5Y cells have served as a human-relevant platform to investigate the neurotoxic and developmental effects of pharmaceuticals and environmental compounds, including antiepileptic drugs and other widely prescribed medications (Table 1, f, g), and have also been proposed as a model for neuronal aging (Strother et al., 2021). In parallel, NT2/D1 cells have been used in neurodevelopmental neurotoxicity screening assays (Taylor et al., 2019), and the investigation of astrocyte reactivation (Balint et al., 2024). The integration of high-content imaging, electrophysiology, and omics approaches expands the utility of dissociated systems for screening and therapeutic target discovery (Piwecka et al., 2023).

Extensions of dissociated cultures to multicellular co-culture formats have enabled modeling of neurovascular interactions. Blood-brain barrier (BBB)-like systems composed of neurons, astrocytes, pericytes, and endothelial cells reproduce aspects of barrier permeability, inflammation, and transporter function, supporting studies on neurotoxicity in drug delivery (Table 1, h–k). Parallel advances in bioengineering, including biomaterial scaffolds, nanofiber matrices, and 3D bioprinting, have enabled more structured neuronal growth and extended viability (Tang-Schomer et al., 2018; Fantini et al., 2019). Hybrid co-culture models integrating neurons with glial or endothelial cells reconstitute elements of the neurovascular unit (Stone et al., 2019; Peng et al., 2021) and 3D LUHMES constructs for dopaminergic toxicity testing (Smirnova et al., 2016) illustrate the expanding scope of dissociated systems.

However, despite these advances, dissociated primary or immortalized human neuronal cultures remain limited for neurodevelopmental disorders such as autism or epileptic encephalopathies because these conditions arise from disruptions in neuronal maturation, subtype specification, and network-level excitatory-inhibitory balance, features that simple dissociated cell cultures cannot reliably recapitulate as they lack sufficient network architecture and often exhibit altered inhibitory excitation-inhibition balance and hypersynchronous activity (Wagenaar et al., 2006; Li et al., 2007). Moreover, the long developmental timescales necessary to model neurodevelopmental disorders exceed the maturation capacity of traditional dissociated cultures.

Consequently, the field has largely shifted toward iPSC-derived neurons and organoids, which enable capturing human developmental trajectories and circuit formation. The development of stem cell- and iPSC-derived neuronal cultures offers the possibility to generate patient-specific neurons and glia that recapitulate genetic backgrounds and disease-specific phenotypes. These approaches extend the translational approach of *in vitro* models by integrating human cellular diversity with scalable and standardized culture systems, providing a platform for mechanistic studies, human-specific validation, and question-driven investigation. Together, these features make iPSC-derived models particularly suitable to explore developmental processes and circuit-level dysfunctions.

### 3.2 Stem cell-derived model systems

#### 3.2.1 Historic evolution from embryonic to induced pluripotent stem cells: strengths and limitations

The hunt for cells carrying the ability to differentiate into any human tissue type in order to provide tissue and organs “off-the-shelf” for medical purposes dates back to well beyond the 20th century. This widely sought-after potential was first found in embryonic stem (ES) cells, initially isolated from mouse blastocysts over 40 years ago (Evans and Kaufman, 1981). These ES cells could be perpetually propagated in laboratory conditions while retaining the potential to differentiate into various mature somatic phenotypes upon exposure to specific morphogens. This breakthrough revolutionized embryonic developmental research and held promise for generating tissue “on demand” for therapeutic applications if stem cells could also be isolated from human blastocysts (Rippon and Bishop, 2004). Human pluripotent ES cells were first isolated in 1998 (Thomson et al., 1998) from spare embryos intended for *in vitro* fertilization (IVF) and cultured *in vitro* for up to 5 months without losing their capacity to differentiate into trophoblasts and derivatives of all three embryonic germ layers. Moreover, neural progenitors derived from human ES cells exhibited the ability to differentiate into all three neural lineages, namely oligodendrocytes, astrocytes, and mature neurons (Reubinoff et al., 2001). However, the use of human-derived ES cells sparked contentious debates concerning the ethical use of embryos for research and regulatory concerns over human cloning research (Orive et al., 2003). This debate intensified when researchers shifted from spare IVF embryos to somatic cell nuclear transfer (Wilmut et al., 2002), leading to swift prohibitions in many countries (Andorno, 2002). Thus, ethical considerations remained unresolved, given the morally and politically charged nature of obtaining human ES cells.

To circumvent these ethical concerns, a groundbreaking protocol was developed to induce pluripotency in adult mouse somatic fibroblast cultures by introducing transcription factors Oct3/4, Klf4, Sox2, and c-Myc (Takahashi and Yamanaka, 2006). These so-called induced pluripotent stem cells (iPSCs) could also be generated from human dermal fibroblasts using the same four transcription factors (Takahashi et al., 2007), opening new avenues for research. iPSCs offer numerous advantages, including easy isolation from accessible adult cell types like skin, blood, and urine cells (Raab et al., 2014). They retain the patient's genetic and epigenetic background, allowing for the manifestation of disease-relevant phenotypes. This enables patient-specific disease modeling and investigation of disease pathogenesis, progression, and drug responses.

Generally, various factors influence the variability of reprogramming efficiency and cellular phenotype (Volpato and Webber, 2020), including the induction method, inter-individual genetic variation, and cell source origin tissue (Scesa et al., 2021). Low efficiency, variability, and partial reprogramming are prominent issues during iPSC induction (Yamanaka, 2009; Hu et al., 2010). Additionally, the initially frequently used retroviral approach of delivering reprogramming factors often results in abnormal gene expression, altered differentiation, or malignant transformation, leading to immunogenicity and rejection of iPSCs by the donor organism (Ghosh et al., 2010; Gore et al., 2011; Zhao et al., 2011). As a result, efforts have been made to develop reprogramming protocols free of retroviral factors, utilizing methods such as delivery with small molecules (Kim et al., 2020), synthetic modified mRNA (Warren et al., 2010), non-integrating adenoviruses (Stadtfeld et al., 2008), or Sendai viruses (Fusaki et al., 2009). Another challenge is the retention of some residual epigenetic methylation profiles from the somatic tissue of origin, combined with *de novo* aberrant methylation, leading to distinct methylation profiles when comparing iPSCs to ES cells or even origin somatic cells (Lister et al., 2011; Ohi et al., 2011). Additionally, copy number variations arising after reprogramming have been reported in iPSCs (Hussein et al., 2011; Popp et al., 2018). A significant proportion of variability in iPSCs can be attributed to laboratory-specific microenvironmental contexts and protocols (Newman and Cooper, 2010), highlighting the need for standardized lines, protocols, and genetic screenings (Pantazis et al., 2022).

In addition, genomic editing techniques such as CRISPR/Cas9 (Jehuda et al., 2018; McTague et al., 2021) facilitate the investigation of the role of genetic disease-associated variants. The pluripotent capacity of iPSCs to differentiate into all three neural lineages, akin to human ES cells, allows for the examination of developmental processes, differentiation, migration, and maturation (Meijer et al., 2019; Autar et al., 2022). Furthermore, their ability to be generated in large numbers makes iPSCs suitable for use as high-throughput drug screening and toxicity testing platforms (Elitt et al., 2018; Chang et al., 2020).

In conclusion, iPSCs possess valuable and promising features such as easy accessibility, retention of patient-specific genetic background, and developmental properties. Nevertheless, issues such as cell maturity and cell-type specificity must be addressed for successful translational approaches. Historically, differentiation into mature, functional neurons required laborious differentiation protocols of several months (Nicholas et al., 2013), making their use time-consuming. However, recent advances in differentiation and maturation protocols have substantially improved the applicability of iPSC-derived neural cells in human disease modeling, making them a viable option for translational research.

#### 3.2.2 Applications of stem cell models in human neuroscientific research and drug development

Recent iPSC culturing methods yield functionally, genetically, and morphologically mature neurons in less time than before (Gunhanlar et al., 2017; Nadadhur et al., 2017). Neuronal maturation is further enhanced by co-culture with astrocytes or growth in three-dimensional environments, including hydrogels, bioengineered scaffolds, and self-organizing spheroids and organoids, which collectively promote structural organization and functional development (Table 2, a–d). Brain organoids have been shown to reliably generate a rich diversity of cell types representing the human cortex (Velasco et al., 2019) or cerebellum (Atamian et al., 2024), and establish mature properties like dendritic spines and spontaneous electrical activity (Quadrato et al., 2017). Additionally, they have been used to study the evolution of the human brain (Mora-Bermúdez et al., 2016) and the physics of human brain folding (Li et al., 2017).

**Table 2:** Application examples for section 3.2 on stem-cell derived model systems.

Ref.	Model system	Cell type/source	Application/ disease	Study focus	Key findings	References	Article type
a	2D neuron/astrocyte co-cultures	hiPSC-derived	Neurodevelopment	Role of human astrocytes in neuronal maturation and synaptic function	Maturation of glutamatergic synapses depends on astrocyte-lineage cells; astrocytes actively modulate synaptic transmission and promote network maturation	Klapper et al., 2019; Hedegaard et al., 2020	Original article
b	Cortical neurons in 3D hydrogel cultures	hiPSC-derived	Tauopathies	Neuronal maturation and tau splicing in 3D culture	Developmental tau splicing switch enables adult tau isoform expression; 3D cultures show higher cell viability and increased markers of neuronal and synaptic maturation	Miguel et al., 2019; de Leeuw et al., 2021	Original article
c	3D neural stem cell neurospheres	hiPSC-derived	Neural differentiation and proliferation	Comparison of embryoid body-based vs. adherent induction protocols	EB-based induction generates superior proliferation and differentiation capacity compared to adherent protocols	Pauly et al., 2018	Original article
d	3D cerebral organoids	hESC-/hiPSC-derived	Neurodevelopment	Combining self-organizing organoids with bioengineered scaffolds to improve reproducibility and architecture	Improved cortical development, radial organization and neuronal migration	Lancaster et al., 2017	Original article
e	2D neuronal cultures	hiPSC-derived	Alzheimer's disease	Role of ApoE4 in tau pathology, amyloid-beta production, and neuronal vulnerability	ApoE4 expression resulted in a gain of toxic effects regarding tau and amyloid-beta	Wang et al., 2018	Original article
f	2D basal forebrain cholinergic neuronal cultures	hiPSC-derived	Alzheimer's disease	Cell-autonomous neuronal insulin signaling and its impact on AD pathology	Familial AD PSEN2 mutation model, potential physiological role for insulin as a mediator of resilience by counteracting specific metabolic and molecular features of AD	Moreno et al., 2018	Original article
g	3D cerebral organoids	hiPSC-derived	Neurodevelopment	Air-liquid interface culturing for improved viability and development of cerebral organoids	ALI culturing resulted in improved survival, axonal outgrowth and functional circuit output	Giandomenico et al., 2019	Original article
h	3D brain organoids	hiPSC-derived	Neurodevelopment	Methodological advances, capabilities and limitations of brain organoids	Organoids recapitulate many molecular, cellular, structural, and functional features of early human brain development, but distinct cortical layering, gyrification, and circuitry are incompletely modeled	Qian et al., 2019	Review
i	3D cortical brain organoids	hiPSC-derived	Neurodevelopment	Emergence and maturation of functional neuronal network activity in human cortical organoids	Organoids exhibit progressive, long-term increases in spontaneous electrical activity and complexity	Trujillo et al., 2019	Original article
j	Microglia cultures	hiPSC-derived	Neurodevelopment; Neuroinflammation	Microglial differentiation and maturation of iPSC-derived microglia	Comparison of differentiation protocols, forward programming approaches, and outcomes on microglia phenotype	Wurm et al., 2021; Csatári et al., 2024	Review
k	2D astrocyte cultures	hiPSC-derived	Neurodevelopment	Development of a rapid differentiation protocol for mature human astrocytes	Differentiation protocol results in mature astrocytes, comparable to human fetal astrocytes	Voulgaris et al., 2022	Original article
l	2D dopaminergic neuronal cultures	hiPSC-derived	Parkinson's disease	Modeling oxidative stress and vulnerability in PD-relevant dopaminergic neurons	DA neurons from G2019S-iPSCs exhibited PD hallmarks and models early PD phenotype	Nguyen et al., 2011	Original article
m	2D cortical neurons	hiPSC-derived	Alzheimer's disease	Modeling tau aggregation and testing compound efficiency	Expression of TAU-P301L did not trigger tau aggregation, but seeding with preformed tau aggregates did; suitable for compound screening	Verheyen et al., 2015	Original article
n	iPSC-based CNS disease models	hiPSC-derived	Neurodevelopment; Neurodegeneration	Synaptic dysfunction mechanisms across developmental and degenerative CNS disorders	Utility of iPSC-based models for mechanistic studies and therapeutic development in synaptopathies	Taoufik et al., 2018; Trombetta-Lima et al., 2021	Review
o	2D neuronal cultures	hiPSC-derived	Schizophrenia	Modeling cellular and molecular defects in SCZD neurons	iPSC-derived SCZD neurons display schizophrenia hallmarks, partially alleviated by antipsychotics	Brennand et al., 2011	Original article
p	iPSC-based neuronal models	hiPSC-derived	Huntington's	iPSC-based HD models, screening assays, and disease modeling strategies	HD patient-derived iPSCs provide versatile platforms to study cellular and developmental aspects of HD	Zhang et al., 2016	Review
q	3D neuron/astrocyte co-cultures	hiPSC-derived	Neurotoxicity	Development and characterization of a high-throughput 3D neural culture platform for assessing compound-induced neurotoxicity	3D co-cultures exhibit spontaneous calcium oscillations, 57% of 87 tested compounds showed measurable effects; platform usable as predictive neurotoxicity testing platform	Sirenko et al., 2019	Original article
r	3D forebrain organoids	hiPSC-/hPSC-derived	Neurodevelopment	Positional topography in cerebral organoids using sonic hedgehog gradient	SHH gradients enable dorso-ventral and antero-posterior self-organization	Cederquist et al., 2019	Original article
s	3D cortical brain organoids	hiPSC-/hESC-derived	Neurodevelopment	Evaluating cellular diversity, maturation, and area-specific neuronal identity in cortical organoids	Organoids generate broad cell classes, but fail to recapitulate distinct cortical subtype identities and progenitor maturation; exhibit unique developmental programs distinct from fetal brain	Bhaduri et al., 2020; Tanaka et al., 2020	Original article
t	3D cortical brain organoids	hiPSC-derived	Neurodevelopment; Neurodegeneration	Review of network formation, cytoarchitecture, and cellular maturity in brain organoids	Brain organoids reproduce cortical cytoarchitecture, generate multiple brain regions and cell types, and gradually form complex neural networks	Porciúncula et al., 2021	Review

##### 3.2.2.1 Neurodegenerative disorders

iPSC technologies have become central to modeling neurodegenerative disorders, largely because they enable genetically patient-specific tissues to be generated *in vitro*. Early studies of AD demonstrated that neurons derived from iPSCs of familial AD patients recapitulate hallmark phenotypes such as increased amyloid-beta secretion and altered tau phosphorylation (Yagi et al., 2011; Israel et al., 2012). Subsequent work refined these approaches, showing that the introduction of tau repeat domain mutations yields tau levels comparable to those observed in post-mortem AD brains (Reilly et al., 2017), while 3D culture technologies markedly accelerate amyloid-beta deposition and tau pathology (Kim et al., 2015; Choi et al., 2016). Cerebral organoids platforms further expanded the modeling repertoire: patient-derived organoids develop progressive AD-like changes, including extracellular amyloid-beta and phosphorylated tau aggregation in a spatially organized tissue context (Gonzalez et al., 2018). These models not only serve for phenotype discovery, but also for mechanistic analysis, as illustrated by proteome-wide mapping of early AD-related dysfunction in iPSC-derived neurons (Pomeshchik et al., 2023).

Importantly, iPSC models also enable testing of mutation-specific therapeutic strategies to correct the phenotype (Table 2, e–f), highlighting how iPSCs can support precision medicine approaches by linking molecular phenotypes to therapeutic or pharmacological targets. Yet, sporadic AD remains challenging to model due to heterogeneous etiologies and more subtle phenotypes (Rowland et al., 2018; Penney et al., 2020). Comparative work, for example, 2D vs. 3D iPSC neuronal cultures (Centeno et al., 2018) or cross-validation with iPSC lines from clinically characterized AD patients (Raska et al., 2021) is beginning to clarify how model architecture influences robustness of sporadic disease readouts.

PD research has followed a similar trajectory. Protocols for deriving midbrain dopaminergic neurons from iPSCs reliably capture disease-relevant hallmarks such as alpha-synuclein aggregation, mitochondrial dysfunction, and oxidative stress (Devine et al., 2011; Kriks et al., 2011). Recent omics-driven studies integrate transcriptomic and proteomic profiling to dissect the molecular pathways underlying PD pathology (Krach et al., 2020), while experimental therapeutics can be rapidly screened in these systems. For example, high-content imaging combined with proteomics identified a kinase inhibitor capable of rescuing alpha-synuclein-related phenotypes in patient-derived neurons (Antoniou et al., 2022). Newer 3D dopaminergic organoid systems derived directly from fibroblasts closely capture aged human brain features and extend modeling capabilities to later-life PD pathophysiology, even enabling further maturation by assembling neuron-astrocyte co-cultures (Kim et al., 2025).

Huntington's disease (HD) is another field where iPSCs have proven as a powerful tool. HD iPSC lines generate striatal neurons that manifest huntingtin aggregation, transcriptional dysregulation, and metabolic impairments (Zhang et al., 2010; Choompoo et al., 2021). These phenotypes enable systematic drug screening through the utilization of, for example, HD patient-derived iPSCs for evaluating candidate therapeutics that target early disease processes (Akimov et al., 2021). Together, these studies show how iPSCs bridge cellular pathology with therapeutic development in neurodegeneration.

##### 3.2.2.2 Neurodevelopmental disorders

iPSCs also offer access to early human neurodevelopment, enabling mechanistic studies of disorders that originate during embryogenesis. In Rett syndrome, patient-derived neurons display synaptic and electrophysiological abnormalities, which can be partially rescued through targeted molecular interventions (Marchetto et al., 2010; Tang et al., 2016). Extending beyond 2D systems, organoids generated from Rett patients reveal transcriptomic alterations and network-level hyperexcitability, including epileptiform discharges (Samarasinghe et al., 2021), reinforcing the value of 3D tissue contexts for capturing circuit-level phenotypes. Lissencephaly, a disorder of cortical development, has similarly been modeled using patient-derived iPSCs (Bershteyn et al., 2017). These models uncover disruptions in neuronal migration and differentiation that align with known genetic etiologies such as LIS1 mutations (Shahsavani et al., 2018). Broader developmental insights emerge from studies exploring human-specific features of corticogenesis using cerebral organoids (Otani et al., 2016; Pollen et al., 2019). Sliced and long-term organoid preparations can help overcome limitations such as nutrient diffusion and allow investigation of lamination, network maturation, and oscillatory activity over months (Table 2, g–i). iPSCs have also proven valuable in modeling rare genetic syndromes associated with intellectual disability, autism (Brant et al., 2021), and epilepsy. Patient-derived neurons carrying IQSEC2 mutations show altered synaptic signaling and excitatory/inhibitory balance (Brant et al., 2021), while STXBP1 encephalopathy models reveal impairments in GABAergic synapse function (Ichise et al., 2021). CRISPR-engineered organoids have further enabled the study of complex syndromes such as ataxia and WOREE syndrome (Steinberg et al., 2021), demonstrating how genome engineering and organoid technologies can lead to the elucidation of developmental mechanisms.

##### 3.2.2.3 . Epilepsy and channelopathies

iPSC-derived models have become increasingly informative for epilepsy research, particularly for monogenic channelopathies, where defined molecular defects can be recreated *in vitro*. For example, neurons derived from individuals with SCN8A mutations show distinct changes in sodium current amplitudes and gating kinetics (Tidball et al., 2020), offering a mechanistic explanation for seizure susceptibility. In a model for KCNQ2-Developmental and epileptic encephalopathy using iPSC-derived neurons, important parts of the disease-related changes were due to deficits in maturation, which can be nicely studied in detail using an iPSC-derived model system (Rosa et al., 2025). Reviews summarizing the field emphasize the utility of iPSC models for studying ion channel dysfunction across epilepsies (Javaid et al., 2022; Simkin et al., 2022). Importantly, these systems allow analysis of network-level alterations in excitation/inhibition balance, as demonstrated by cortical networks derived from patient iPSCs (Liu et al., 2019b; Yokoi et al., 2022). Organoid technologies further extend epilepsy models by enabling circuit-level studies. Organoids from developmental epilepsy patients exhibit aberrant rhythmic activity, migratory defects, and transcription dysregulation (Nieto-Estévez and Hsieh, 2020). Dravet syndrome has been modeled using ventral forebrain organoids derived from patient iPSCs, which recapitulate GABAergic interneuron deficits central to disease pathophysiology (Zayat et al., 2023). A hybrid *in vitro* approach also exists by coculturing iPSC-derived cortical neurons with human epileptic brain biopsy tissue on multielectrode arrays that reveal patient-specific seizure signatures and functional coupling (Hu et al., 2023), providing a unique translational link between laboratory models and clinical pathology. Focal cortical dysplasia has also been modeled using iPSCs to investigate genes regulating proliferation, adhesion, and apoptosis during cortical development (Marinowic et al., 2020). These studies collectively illustrate how iPSCs can map pathogenic cascades from molecular mutations to network hyperexcitability.

##### 3.2.2.4 Cellular interactions, microenvironment, and barrier models

Beyond neuron-centric models, iPSCs allow the generation of major non-neuronal cell types relevant to neurological disease. Protocols for producing functional astrocytes and microglia-like cells facilitate investigation of cellular interactions (Table 2, j, k). Co-cultures of iPSC-derived glial cells have revealed synergistic degradation of pathological protein aggregates such as alpha-synuclein and amyloid-beta, as well as active modulation of inflammatory signaling across the BBB via defined molecular pathways (Rostami et al., 2021; Kim et al., 2022). iPSC-derived vascular cells additionally support the construction of human BBB models, enabling direct comparison with animal-derived models (Di Marco et al., 2020). Incorporation of vascular structures into organoids further enhances nutrient diffusion and tissue maturation (Matsui et al., 2021; Kook et al., 2022), representing an important step toward physiologically relevant disease models.

Advances in differentiation protocols, 3D culture technologies, and CRISPR-based editing have made iPSCs a cornerstone of preclinical research. The ability to model protein aggregation, mitochondrial dysfunction, oxidative stress, and synaptic alterations aligns these systems with major pathogenic pathways across neurological diseases (Table 2, l–n). Their scalability further positions iPSC models as platforms for high-throughput drug screening and neurotoxicity testing with promising translational implications (Table 2, o–q).

Importantly, iPSC-based applications are now transitioning toward clinical applications. Transplantation of iPSC-derived retinal pigment epithelial cells has demonstrated long-term graft survival for the first time, alongside increased retinal thickness and slower visual decline in retinitis pigmentosa patients without adverse effects (Hirami et al., 2023), while recently, a phase I/II trial of iPSC-derived dopaminergic progenitors for PD reported stable graft survival, dopamine production, lack of tumor formation, and functional improvements in most participants over a 2-year period (Sawamoto et al., 2025). These developments show a rapidly evolving clinical use of autologous and allogenic iPSC-based grafts and growing interest in applying iPSC and organoid technology to neurodegenerative diseases such as PD and AD (Wang et al., 2022; Hui and Yamanaka, 2024). Moreover, the combination of iPSC technologies with CRISPR/Cas9 gene correction is driving a new era of personalized cell replacement approaches (Sguazzi et al., 2021), contributing to the overall evolution of stem-cell-based therapies for neurological disorders (Rahimi Darehbagh et al., 2024).

Despite these promising advances, challenges remain. Self-organizing organoids still exhibit variability in cell type composition and topographical organization, and their lack of functional and physiological vascularization may limit long-term maturation (Table 2, h, r–t). iPSC-derived tissues may also face issues related to tumorigenicity, immunogenicity, and heterogeneity in translational applications (Zhong et al., 2022). These pitfalls highlight the need for continued refinement of bioengineering strategies and guided differentiation.

As these advances progress, integration with emerging bioengineered systems offers complementary opportunities to address remaining limitations. In particular, microfluidic and microengineered “organ-on-a-chip” systems provide precise control over fluid dynamics, nutrient delivery, and microenvironmental cues, enabling investigation of specific interfaces. These platforms serve as complementary, technically highly sophisticated tools, facilitating the detailed in-depth analysis of specific structural and functional features of the central nervous system.

### 3.3 Microengineered and microfluidic models

#### 3.3.1 Emergence of the brain-on-a-chip: technical strengths and constraints

In recent years, the concept of Organs-on-Chips (OoCs) has been widely implemented to generate artificial organs grown directly on microfluidic chips designed to control cellular microenvironments and maintain tissue-specific functions (Leung et al., 2022). This technology represents a promising platform for exploring specific structural and functional aspects of brain physiology. By providing precise experimental control and high reproducibility, it supports mechanistic and detailed investigations of defined phenomena, such as the neurovascular unit, complementing existing experimental approaches (NC3Rs, 2021).

The proximity of engineering and neuroscience increased dramatically in the early 21st century, driven by advances in microfabrication, microfluidics, and materials science that enabled very precise control over cellular microenvironments. The foundation was laid decades earlier with multi-electrode arrays (MEAs), first demonstrated with recordings from cultured embryonic chick heart cells (Thomas et al., 1972). After these first promising steps, the integration of microfluidic systems in the following decades revolutionized the field by allowing precise spatiotemporal control of biochemical gradients, mechanical forces, and cellular architectures. Toward the brain-on-a-chip (BoC) technique, a pioneering study developed a compartmentalized microfluidic platform that could fluidically isolate CNS axons from their cell bodies, enabling breakthrough studies in axonal biology and regeneration (Taylor et al., 2005; Molina-Martínez et al., 2022). This engineering toolkit transformed neuroscience research: BoC platforms emerged that could recreate complex neural architectures and, in part, model neuron-glia interactions (Park et al., 2009) and allowed the development of a multicompartment setting to study complex diseases like epilepsy (Pelkonen et al., 2020). The development of transparent, biocompatible materials combined with advanced imaging techniques enabled real-time visualization of neural network formation and activity. By integrating electrical recording capabilities, perfusion systems, and 3D tissue culture within microscale devices, engineers provided neuroscientists with powerful new tools to investigate fundamental questions about neural development, synaptic plasticity, and disease mechanisms. Most commonly, human iPSC-derived cells are used for these models, but primary cells or organotypic brain cultures can also be employed (Liu et al., 2019a).

Despite these strengths, critical challenges remain before the technology can be widely adopted for routine use. These include further characterization and validation to establish that the technology is suitable for its intended application. Several limitations associated with OoCs stem from challenges related to iPSC technology, as discussed in detail above. Another limitation is that OoCs cannot fully recapitulate the structural and functional complexities of organs and tissues, since they typically incorporate only a limited number of cell types or are capable of assessing just limited functional parameters. This constraint is especially challenging for the brain, with its highly diverse cell types capable of specific long-range connections and complex local cellular interactions. Moreover, the generation of these complex microsystems requires not only specific knowledge in engineering processes, but also the corresponding equipment, which is currently not feasible for a wide range of laboratories. Nevertheless, OoCs have laid the groundwork for precision modeling of neurological diseases.

#### 3.3.2 Application of BoCs in human disease modeling and drug development

OoCs are microengineered platforms designed to recreate key structural and functional aspects of human tissues by interfacing living cells with precisely controlled physical and biochemical environments. They are not intended to fully recapitulate the complexity of the human brain *in vivo*, but instead to enable focused investigation of selected biological interfaces and mechanisms (Cerutti and Brofiga, 2024). Since their development, they have been widely adopted as alternatives to animal experiments for disease modeling, drug development, toxicology, and personalized medicine (Ingber, 2022).

##### 3.3.2.1 The blood-brain-barrier: drug delivery, disease, and neurovascular biology

The BBB has become one of the most impactful applications of BoC technology. Early BBB-on-chip models demonstrated selective permeability and tight junction formation (Booth and Kim, 2012), while more recent systems have achieved *in vivo*-like barrier function by integrating hiPSC-derived endothelial cells with microglia, astrocytes, and pericytes under dynamic flow (Pediaditakis et al., 2022). Recent systems reported expression of tight junction proteins and efflux transporters, selective transcytosis of peptides and antibodies as observed *in vivo*, establishing very robust platforms for the development and validation of drug and antibody delivery strategies into the brain (Park et al., 2019).

Increasingly complex BBB and neurovascular unit models now also incorporate neurons and microglia to capture higher-order functional interactions. A human organotypic microphysiological system composed of endothelial cells, pericytes, glia, and cortical neurons was reported to maintain BBB permeability near *in vivo* levels and to reproduce key features of neuroinflammation upon exposure to TNFalpha (Pediaditakis et al., 2022). These complex systems are now also suitable to model pathogen entry into the brain, for example, during the pathogenesis of fungal meningitis, where fungal pathogens have been shown to hijack transcytosis-mediated mechanisms at the BBB (Kim et al., 2021).

Beyond disease mechanisms, BBB-on-chip platforms are widely used to investigate drug transport and delivery techniques, including nanocarriers (Lee et al., 2020). Standardized protocols for vascular permeability analysis have been established to support reproducibility (Hajal et al., 2022). Collectively, these studies position the BBB-on-chip as a foundational technology for understanding neurovascular biology and evaluating CNS drug delivery.

##### 3.3.2.2 Disease modeling and personalized medicine

BoC platforms have also been successfully applied to model neurological diseases at different levels, leveraging patient-derived hiPSCs to enable personalized approaches. Neurodegenerative diseases such as AD and PD have been studied using these systems, which can capture disease-relevant and patient-specific genetic backgrounds and neuroinflammatory responses (Yoon et al., 2021; Cerutti and Brofiga, 2024). For example, a substantia nigra-on-a-chip including dopaminergic neurons, astrocytes, microglia, pericytes, and endothelial cells under fluid flow recapitulates key pathological features of PD after exposure to alpha-synuclein fibrils, demonstrating the platform's utility for studying cell-cell interactions as well as for identifying and validating therapeutic targets in human synucleinopathies (Pediaditakis et al., 2021).

BoC systems have also enabled modeling of network-level disorders, including epilepsy. Platforms featuring spatially separated but functionally connected neuronal circuits, often integrated with microelectrode arrays, allow real-time monitoring of pathological activity, seizure propagation, and drug responses (Liu et al., 2019a; Pelkonen et al., 2020). In parallel, patient specificity has become a defining feature in the field. For example, 3D spheroids derived from patient glioblastoma specimens were cultured on a brain cancer chip in order to evaluate specific responses to bevacizumab and temozolomide (Akay et al., 2018), demonstrating the potential of the system to use it for evaluating and tailoring drug combinations to individual patients.

Recent emerging models extend personalized disease modeling beyond the brain by incorporating communication between different organs. Gut-brain axis chips have shown that gut microbiota-derived metabolites and extracellular vesicles influence neuronal growth, maturation, and synaptic plasticity, providing mechanistic insights into how systemic factors could contribute to disease susceptibility and progression (Kim et al., 2024).

##### 3.3.2.3 Toxicology and systems-level drug evaluation

Toxicology and safety assessments, particularly in contexts where animal models fail to correctly reproduce human-specific responses, can be effectively performed in OoC systems. Integrating multiple neural and vascular cell types, these systems allow controlled exposure to environmental toxins, chemical agents, or new drugs, while capturing complex human cellular interactions. 3D tetra-culture BoC systems have been developed, incorporating BBB components, neurons, astrocytes, and microglia to screen for organophosphate toxicity (Koo et al., 2018). Similarly, 3D hiPSC-derived GABAergic neuron-astrocytes chips have been used to assess malathion-induced neurotoxicity and evaluate the efficacy of butyrylcholinesterase post-treatment (Liu et al., 2020b).

Toxicology studies also increasingly benefit from multi-organ integration, for example, by combining a BBB model with liver and cortical spheroids derived from the same donor, allowing investigation of compound permeation, hepatic metabolization, and downstream effects on the brain (Koenig et al., 2022). Such systems are particularly interesting for compounds where neurotoxicity is mediated by metabolites rather than parent molecules. BBB-tumor models further bridge toxicology and therapeutic evaluation, enabling the investigation of chemically opening the BBB for better drug delivery targeting brain tumors (Seo et al., 2022).

Collectively, these studies highlight how BoC platforms enable integrated evaluation of toxicity, pharmacokinetics, and efficacy in a human-relevant context. As with all models, BoCs present both strengths and limitations, and their suitability ultimately depends on the biological question asked. However, for specialized mechanistic and personalized insights, they provide a powerful framework for advancing our understanding of human neurological disease mechanisms and therapeutic interventions.

While BoC platforms excel at dissecting specific cellular and molecular interactions, research questions focused on network-level dynamics, intact circuitry, or complex neuron-glia interactions require models that retain native tissue architecture. In this context, human brain slices offer a powerful approach for investigating phenomena that are reductionist or inaccessible in other platforms, perfectly complementing the array of human-based model systems with a platform that focuses on the investigation of system-level research questions.

### 3.4 Acute and cultured brain slices

#### 3.4.1 Evolution of brain slice methodology: advantages and disadvantages

Acute brain slices are a valuable tool in neuroscientific research when the anatomical and functional integrity and the three-dimensional cytoarchitecture of the brain region need to be conserved. Historically, most of this work has been conducted in rodent brain slices, where these preparations have been essential for investigating neuronal connectivity, excitation-inhibition balance, and circuit interactions (Zolnik et al., 2017; Yang et al., 2022). Acute brain slices also preserve all cell types within the corresponding brain region and maintain their specific interplay (Schipke et al., 2002) and they can be obtained at different developmental stages to investigate the maturation of electrophysiological and morphological properties (Kroon et al., 2019; Ciganok-Hückels et al., 2022). A further advantage of rodent acute slices is the availability of adult and transgenic animals, which enables the investigation of mature neuronal properties and age-related conditions (Campbell et al., 2012).

With the growing need for translational models, acute slices have also been prepared from human surgical specimens, which preserve the donor's genetic background and individual properties of the tissue donor (Campbell et al., 2012; Liu et al., 2020a). The first human slice recordings were reported in 1973 (Kato et al., 1973), establishing an important bridge toward human-specific neurophysiology. Human slices have since been used to characterize neuronal subpopulations, morphoelectrical properties, and ion channel or membrane features relevant to human features such as intelligence (Goriounova et al., 2018; Lee et al., 2023).

Across both species, acute slices allow precise control of environmental parameters, such as temperature or ion and modulator concentrations. Variables can be easily manipulated, and the ensuing effects can be investigated, for example, by the application of pharmacological agents (Monni et al., 2022) or even combined with modulation of the metabolic state to unmask state-dependent effects (Doi and Ramirez, 2010; Layer et al., 2021). High-resolution imaging approaches, such as 2-Photon or electron microscopy (Ding et al., 2009; Borges-Merjane et al., 2020), are well established in rodent slices, and while first attempts have already occurred in human material decades ago (Cragg, 1976), they are now increasingly applied to human tissue. Rodent and human slices have been shown to preserve both physiological and pathological network oscillations, including disease-relevant high-frequency activity, enabling direct comparison between non-pathological and epileptogenic tissue as well as integration with *in vivo* electrophysiological EEG recordings (Table 3, a–e). These advantages have contributed to the widespread use of acute slices, predominantly rodent, but increasingly human, for studying a broad spectrum of neurological diseases, including neurodegeneration, epilepsy, and brain tumors (Table 3, f–h).

**Table 3:** Application examples for section 3.4 on acute and cultured brain slices.

Ref.	Model system	Species	Application/ disease	Study focus	Key findings	References	Article type
a	Acute cortical slices	Human	Neurophysiology	Characterization of theta and gamma oscillations in human neocortical slices	Slices generate theta and gamma oscillations dependent on intact synaptic activity, differs from epileptiform activity	Florez et al., 2015	Original article
b	Acute hippocampal slices	Human	Epilepsy	High-frequency oscillations and cellular correlates	HFOs associated with distinct population activity, physiological and pathological HFOs	Alvarado-Rojas et al., 2015	Original article
c	Acute cortical slices	Human	Epilepsy	Ultrastructural analysis of synaptic circuitry in dysplastic human neocortex	Cortical dysplasia induces heterogeneous alterations in excitatory and inhibitory synaptic density; identification of abnormal synaptic alterations	Alonso-Nanclares et al., 2005	Original article
d	Acute cortical slices	Human	Epilepsy	Identification of neuronal subtypes with epilepsy-associated transcriptomic alterations	Epilepsy-related transcriptomic changes are subtype-specific, affecting distinct excitatory and inhibitory neurons; strong dysregulation of glutamatergic signaling	Pfisterer et al., 2020	Original article
e	Acute hippocampal slices	Human	Epilepsy	Cellular and network mechanisms underlying the transition from interictal to ictal activity	Pre-ictal discharges emerge in subiculum prior to seizures, preceded by pyramidal neuron firing; recurrent pre-ictal activity triggers seizure onset	Huberfeld et al., 2011	Original article
f	Cultured organotypic cortical slices	Human	Glioblastoma	Patient-specific tumor cell invasion and migration	Significant inter-patient variability in tumor cell migration; EGFR inhibition selectively reduces invasion in EGFR-amplified tumors	Parker et al., 2013	Original article
g	Cultured organotypic cortical slices	Human	Neurodevelopment; Epilepsy	Modeling genetic epilepsy in the developing human cortex using long-term organotypic slice cultures	Long-term viable human fetal cortical slices retain cortical architecture; STXBP1 haploinsufficiency impairs synaptic function and reduced synaptic density	McLeod et al., 2022	Original article
h	Cultured organotypic cortical slices	Human; Mouse	Neurodegeneration	Modeling initiation and spreading of alpha-synuclein pathology in intact brain tissue	Seeded aggregation induces alpha-synuclein inclusions in murine and human brain slice cultures, depends on seed type and genetic background	Barth et al., 2021	Original article
i	Cultured organotypic hippocampal slices	Mouse	Neurodegeneration	Modeling seeded amyloid-beta aggregation in living brain tissue	Seeded amyloid-beta deposition occurs in living hippocampal slice cultures upon exposure to brain extracts from aged APP transgenic mice and synthetic amyloid-beta	Novotny et al., 2016	Original article
j	Cultured organotypic brain slices	Rat	Epilepsy	Modeling epileptiform activity and neuroinflammation	Serum-deprived organotypic slices develop spontaneous epileptic-like activity	Magalhães et al., 2018	Original article
k	Acute and cultured organotypic cortical slices	Mouse	Traumatic brain injury; Epilepsy	Effects of prostaglandin E2 and tetrodotoxin on network excitability	Acute PGE2 reduces excitatory synaptic transmission post-synaptically; long-term PGE2 or TTX exposure induces hyperexcitable networks	Koch et al., 2010	Original article
l	Cultured organotypic hippocampal slices	Rodent	CNS diseases	Use of hippocampal slice cultures to study disease mechanisms, neurotoxicity, and therapeutic interventions	Slice cultures allow long-term study of physiological and pathological processes, can be combined with advanced techniques, and are useful for evaluating compound effects and translational comparisons	Noraberg et al., 2005	Review

However, despite the many advantages of acute brain slices, certain limitations must be considered. Brain slices are usually created at a thickness of several hundred micrometers, meaning they lose their *in vivo* context and lack complex network interaction and inputs from other brain regions. Moreover, tissue damage and mechanical trauma due to the slicing process can alter the properties of neuronal morphology, connectivity, and function (Lipton, 1985). Furthermore, the formation of a glial scar by reactive gliosis at the site of slicing was reported (Takano et al., 2014). There are discussions about whether brain tissue should be sliced at ice-cold temperatures to avoid cellular degradation and excitotoxicity as much as possible (Wassmann et al., 1998), or if this approach leads to alterations such as spine loss and synaptic remodeling compared to slicing at physiological temperatures (Eguchi et al., 2020). General properties of brain slices may show high variability according to age and brain region from which they were obtained. Moreover, oxygen levels and aCSF composition remarkably influence slice viability and electrophysiological properties (Huchzermeyer et al., 2008; Hájos and Mody, 2009), highlighting the need for standardized protocols. One major limitation of both human and rodent acute brain slices is their finite lifespan, usually limited to several hours, with declining viability. This negates the study of long-term processes, such as plasticity or chronic disease states, and does not allow for long-term genetic or pharmacological manipulation. Notably, many efforts have been made in recent years to extend the lifespan of acute brain slices to several days, for example, by elaborate incubation systems (Wickham et al., 2018) or protective slicing solutions (Ting et al., 2018b), which is of utmost importance.

Organotypic brain slice cultures (oBSCs) offer all the advantages of acute brain slices, namely preserved three-dimensional cytoarchitecture, maintained cell type composition and genetic background of the donor tissue (Duff et al., 2002), and controlled environmental parameters. Additionally, they extend the lifespan of these slices to several weeks, enabling an experimental timeframe for genetic manipulation or long-term application of pharmacological agents or electrical stimulation. Their development originated in rodent tissue, beginning with the cultivation of embryonic nervous tissue (Harrison, 1906) and further refined by Gähwiler's roller tube technique using early post-natal rat slices (Gahwiler, 1981). In this method, tissue slices are embedded in a plasma clot, placed on a coverslip, and rotated slowly in nutrient medium, promoting gas exchange and even nutrient distribution while substantially flattening the tissue. Although this method produced very thin cultures (2–3 cell layers), their long-term stability enabled extensive morphological and physiological studies (Klostermann and Wahle, 1999; Dupont et al., 2006). However, as the roller tube technique leads to tissue thinning and, therefore, partial loss of spatial three-dimensional organization, this method could not be favored for electrophysiological and developmental studies where the original configuration of the *in vivo* tissue is required.

To address this, Stoppini et al., modified the technique for oBSCs further in 1991 (Stoppini et al., 1991), describing a cultivation method for rodent hippocampal slices on semi-porous membranes on an air-liquid interface that largely preserved the original form of the brain slice. Due to the limited diffusion of both oxygen and nutrients, slices for this culturing method are usually sliced at a thickness of 200–400 μm, however, elaborate perfusion techniques to maintain slices up to 600 μm thick have been reported (Rambani, 2010). Both methods demonstrated close post-natal alignment with *in vivo* development (Bak et al., 2024b) and have been shown to maintain cell morphology and physiology (Humpel, 2015). Therefore, this system has been used successfully as a model for neurodegeneration, epilepsy, brain damage, neuroprotection, and neurorepair (Table 3, i–l).

A majority of studies prepare oBSCs from neonatal rodents around P7, as this time point marks the peak of the brain growth spurt, which is already reached at birth in humans (Dobbing and Sands, 1979), while still benefiting from the plasticity and, therefore, viability of developing tissue. oBSCs from mature animals have been proven to be much more challenging to maintain, but viability can be improved by reduced slice thickness and mild hypothermic culturing conditions (Mewes et al., 2012). Nevertheless, oBSCs from post-natal rodents undergo development *in vitro* and are, therefore, an ideal model system to study plasticity, circuit maturation, and brain development in general (Johnson and Buonomano, 2007; Hobbiss et al., 2018). This development, however, also leads to different time-dependent structural and electrical culturing phases (Bak et al., 2024b), which should be kept in mind when designing experiments. Moreover, many diseases that occur primarily in the aged brain, such as Alzheimer's or Parkinson's, might not be fully recoverable in this early tissue. Moreover, as with all animal-derived models, the question of the degree of translatability to humans validly arises.

To overcome these limitations, oBSCs can also be created from human specimens. Tissue can be obtained either from “healthy” access tissue during neurosurgical approaches or from resections of diseased parts of the brain, for example, an epileptogenic focus or a tumor. Cultivation of post-mortem tissue has also been utilized (Verwer et al., 2002), however, this approach is tied to difficulties like post-mortem alterations and crucial timing of brain excision. Due to the delicacy and mature nature of human brain tissue, cultivation can be challenging, and many advances have been made to improve culture conditions.

Although the use of chemically precisely defined culturing media led to exceptional viability of brain slices (Ting et al., 2018b), the use of human cerebrospinal fluid (hCSF) as cultivation medium has been shown to improve tissue viability tremendously, and even preserve tonic and phasic network activity up to several weeks *in vitro* (Schwarz et al., 2017). hCSF-cultured adult brain slices have been demonstrated to preserve cortical cytoarchitecture and neuronal electrophysiological properties robustly and to be susceptible to high-throughput labeling by adeno-associated viruses (Schwarz et al., 2019). hCSF has also been suggested as a recording medium for electrophysiological investigations as it increases firing and bursting rates of the neuronal network and, therefore, might be better suited to mimic physiology and disease as observed *in vivo* (Wickham et al., 2020). As hCSF is low in protein and predominantly composed of salts, and artificial CSF could not mimic the neuromodulatory effect, these observations have been speculated to originate from CSF components such as peptides, lipids, or amino acids. Especially, neuromodulators such as neurotransmitters, neuropeptides, and neurosteroids might be essential for neuronal viability and physiology (Bjorefeldt et al., 2018). These long-term culturing approaches have opened possibilities to apply innovative, state-of-the-art methods such as optogenetic modulation and manipulation via viral vectors (Andersson et al., 2016; Butler-ryan and Wood, 2021).

Building on these methodological advances, human acute and organotypic brain slice preparations now offer an opportunity to investigate neurological disease processes in intact and patient-derived circuits.

#### 3.4.2 Applications of human brain slices in circuit physiology and neuropathology

Acute and long-term cultured organotypic human brain slices have become a powerful experimental platform in translational neuroscience by providing direct access to actual, intact human brain matter in physiological and pathological states. Tissue derived from neurosurgical resections or post-mortem tissue can be maintained for days to weeks *in vitro* in a way that preserves electrophysiological activity, cellular composition, and cytoarchitecture. While resected access or “control” tissue from epilepsy or tumor surgeries is often histologically normal and suitable for investigating fundamental human neurobiology or simulating *de novo* disease states, pathological tissue retains features of usual disease hallmarks, both offering unique opportunities. Moreover, recent technical advances, for example optimized tissue handling (Straehle et al., 2023), long-term viability protocols (Bak et al., 2024a; Plug et al., 2024), and compatibility with viral tool and optical imaging (Le Duigou et al., 2018; Ting et al., 2018a), have increased the robustness of these models, positioning human brain slices as an increasingly valuable tool for translational research.

##### 3.4.2.1 Neurodegenerative disorders

Human cortical slices provide a unique platform for studying neurodegenerative processes in an aged human tissue environment. Accessing brain tissue from resective surgeries can be considered mostly healthy in many cases. It can be used to either investigate the healthy human brain per se or to introduce genetic or pharmacological manipulation and observe their impact or course of pathogenesis. This approach has been used to seed alpha-synuclein (Barth et al., 2021) or tau and has proven to be very valuable in studying aggregation diseases such as Alzheimer's or Parkinson's in an aged human environment (Croft and Noble, 2018; Croft et al., 2019).

Treatment of these slices with AD-associated amyloid-beta oligomers triggers hallmark AD features, such as increased hyperphosphorylated tau (Mendes et al., 2018), indicating that human slices can reproduce early molecular cascades of amyloid toxicity. More recent findings highlight the interplay between physiological and pathological amyloid-beta (McGeachan et al., 2025a). In live human slices, amyloid-beta and tau release dynamics vary substantially with donor age and cortical region, suggesting human-specific processes that rodent systems cannot capture. Moreover, increases in physiological amyloid-beta induce synaptic transcript adaptations that may represent compensatory responses, whereas exposure to amyloid-beta-rich AD brain extracts results in synapse loss without transcriptional changes, along with post-synaptic uptake of pathological oligomers (McGeachan et al., 2025a). Also, tauopathies beyond AD can be interrogated in this setting. Using slices from progressive supranuclear palsy (PSP) donors, oligomeric tau was found to accumulate within human synapses and associate closely with astrocytic processes, with astrocytes actively engulfing degenerative synapses (McGeachan et al., 2025b). Parallel approaches combining human slices with reprogrammed neurons have been explored to model cell type-specific vulnerability in neurodegeneration (Kvist, 2021). Together, these studies demonstrate that human organotypic slices offer an unparalleled platform for dissecting synaptic and glial events in protein misfolding diseases.

##### 3.4.2.2 Epilepsy and circuit hyperexcitability

Human brain slices have long been central to epilepsy research, providing direct access to epileptogenic cortical and hippocampal tissue from drug-resistant patients. Such samples reliably retain their disposition for epileptic discharges over days in culture (Eugene et al., 2015) and reveal disease-specific alterations in ion homeostasis and network connectivity. For example, increased expression of the chloride potassium cotransporter KCC2 in surgically resected hippocampi of temporal lobe epilepsy (TLE) patients illustrates species-specific shifts in inhibitory signaling (Karlócai et al., 2016). Acute electrophysiological recordings further indicate that human pyramidal to interneuron synapses possess dramatically more functional release sites than their rodent counterparts, with implications for human-specific patterns of excitation-inhibition balance (Molnár et al., 2016).

Cultured slices provide a test platform for evaluating pharmacological interventions directly in human pathological circuits. A1-adenosine receptor activation suppresses carbamazepine-resistant seizure-like activity in human neocortical slices, underscoring the translational relevance of human tissue-based drug testing (Klaft et al., 2016). Network recordings have revealed multiple forms of synchrony, epileptiform and non-epileptiform, even in tissue derived from non-epileptic patients, highlighting the complexity of human circuit dynamics (Tóth et al., 2018). Optogenetic tools are increasingly applied in this context, from early proof-of-concept demonstrations (Andersson et al., 2016) to recent high-density multielectrode array recordings showing that targeted optogenetic activation can attenuate hyperactivity in human hippocampal slices (Andrews et al., 2024). Reactive astrocytes in TLE slices have been shown to accumulate lipids that enhance neuronal hyperexcitability (Chen et al., 2023), illustrating how the human glial environment shapes seizure susceptibility and how essential organotypic cellular composition is for these investigations. Comprehensive reviews outline both the opportunities and methodological pitfalls associated with this work (Jones et al., 2016; de la Prida and Huberfeld, 2019).

##### 3.4.2.3 Brain tumors and tumor microenvironment

Organotypic human slices preserve the structural and cellular features necessary to model genuine tumor-brain interactions. Microinjection of patient-derived glioblastoma (GBM) cells into cultured cortical slices yields representative patterns of tumor invasion and microtube formation (Ravi et al., 2019; Schneider et al., 2021). This environment supports patient-specific therapeutic testing. For instance, disrupting tumor microtubes profoundly alters GBM network architecture and sensitizes tumors to DNA-damaging therapeutic agents (Schneider et al., 2021). Human slices have also been used to characterize interactions between the tumor and astrocytes or microglia, for example, demonstrating how microglial loss-of-function alters astrocytic phenotypes and cytokine release (Henrik Heiland et al., 2019).

Single-cell transcriptomics have been adapted to tumor engraftment models (Zhang J. et al., 2023), enabling detailed molecular profiling while retaining the advantages of an intact 3D microenvironment. Fluorescently labeled primary GBM cells implanted into adult slices allow for live-cell tracking of invasion patterns and dynamics (Ravin et al., 2023). Beyond modeling exogenous tumor implants, post-mortem and surgical tumor explant cultures maintain native cytoarchitecture and are valuable for studying tumor biology and testing of new drug candidates (Merz et al., 2013; Parker et al., 2017). Collectively, these approaches enable precise oncology research directly in human tissue.

##### 3.4.2.4 Viral neuropathology and host-pathogen interactions

Human organotypic slices have emerged as an ethically accessible model for testing viral tropism and invasion. Fetal and adult tissue cultures replicate fundamental aspects of the pathological processes observed *in vivo*. Herpes simplex virus infection in fetal slices, for example, induces chemokine and cytokine responses, neuronal and astrocytic necroptosis, and spreading infection patterns reminiscent of clinical herpes simplex encephalitis (Rashidi et al., 2024). Similarly, Usutu virus infection mirrors behaviors observed for similar viruses such as West Nile and Zika viruses, including robust replication and defined cell type susceptibility (Bulstrode et al., 2022; Marshall et al., 2024). Adult slices respond to emerging viruses with inflammation, altered electrophysiological signatures, and cytotoxicity, as shown by Oropouche virus infection of human brain slices (Almeida et al., 2021). Recent innovations combine organotypic brain slice culture with machine learning analysis of local field potentials to classify infection states and patterns with high accuracy (Partiot et al., 2024). These features make human slices a promising platform for assessing neurovirulence and screening antiviral strategies with translational relevance.

##### 3.4.2.5 Fundamental human neuropathology and pharmacology

Human organotypic brain slice cultures provide a powerful platform for investigating mechanisms of neuropathology and neuropharmacology. Earlier work showed that cultured human slices reliably reproduce hallmark injury responses, including neuronal degeneration and reactive gliosis following mechanical perturbations (Verwer et al., 2015). More recent advances using post-mortem tissue maintained for up to 6 weeks have extended these capabilities, demonstrating that human slices support experimentally induced demyelination and exhibit physiologically relevant multicellular repair programs (Plug et al., 2024). Imaging innovations further enhance these models, for example, with Third harmonic generation microscopy, allowing label-free, real-time visualization of myelin integrity and axonal structure in viable human slices, providing access to myelin pathology relevant to disorders such as multiple sclerosis (Meijns et al., 2025).

Organotypic brain slice cultures can also be used for pharmacological screening, for example, in neuropsychiatric disorders. Pharmacological perturbation of NMDA receptor signaling with MK-801 in cultured slices alters proteomic pathways relevant to schizophrenia, and regular antipsychotics are able to normalize these changes (de Almeida et al., 2024). Reviews highlight the potential of this platform for studying neuropsychiatric disorders and corresponding potential therapeutics with intact human network context (Qi et al., 2019; Park et al., 2022).

## 4 Discussion

**Figure 3:**
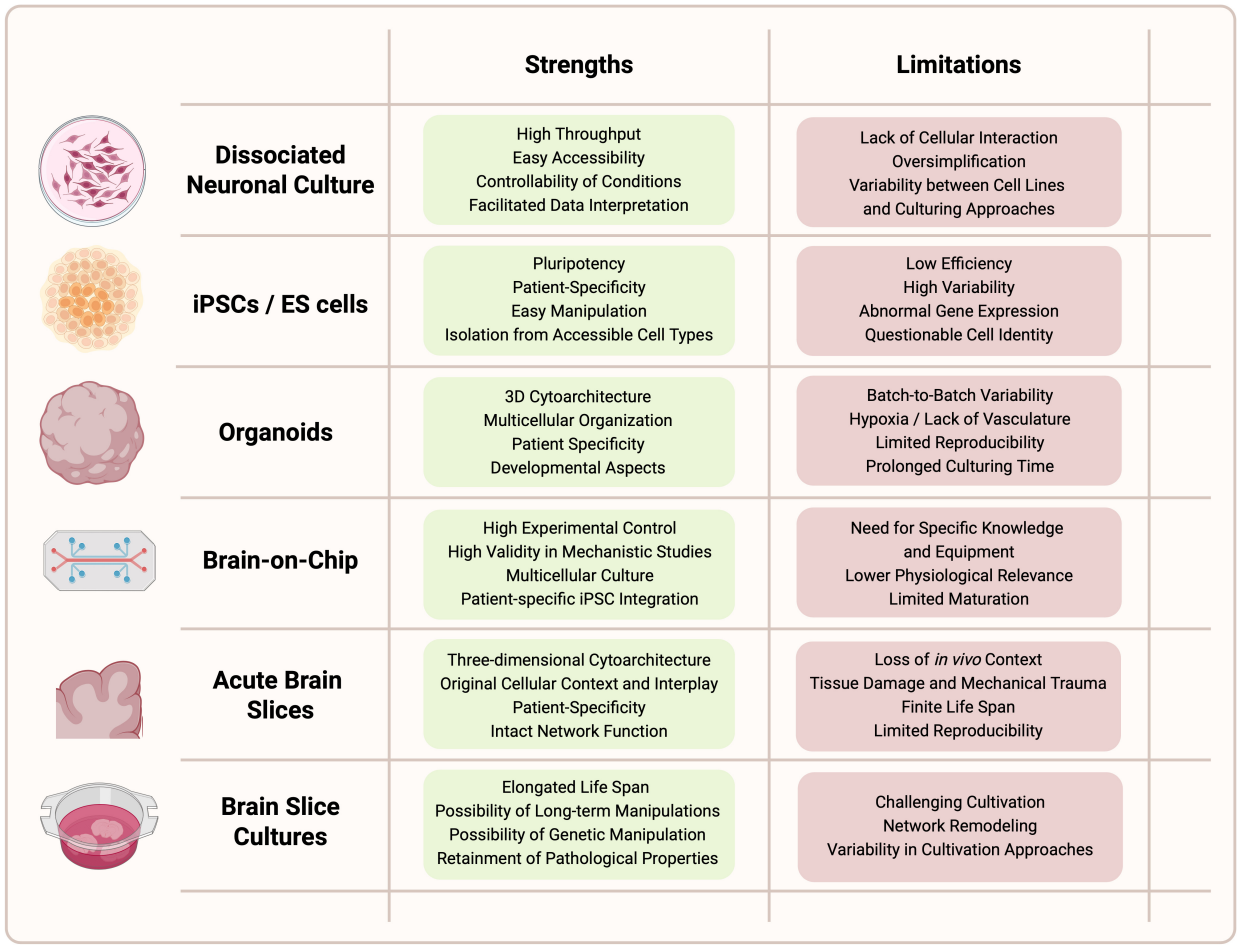
Comparative strengths and limitations of human-based model systems. Overview of individual strengths and limitations of the respective human-based model systems, namely dissociated cultures, iPSCs/ES cells, organoids, brain-on-chip, acute brain slices, and cultured brain slices.

This review illustrated the specific strengths and limitations of human-based model systems currently used in neuroscientific research (Figure 3). However, despite substantial advances in human-based neuroscientific models, several technological, ethical, and regulatory issues continue to impact their full translational potential. Primary human neuronal tissue presents unique challenges that must be addressed to ensure reliability, reproducibility, and broader acceptance.

The use of resected or post-mortem brain tissue remains invaluable for understanding human-specific neurophysiology and pathology, but suffers from major standardization issues (Jones et al., 2016). Protocols for tissue collection, processing, culturing, and analysis vary widely between hospitals and laboratories, impacting reproducibility and comparability. Availability of human tissue is also limited, as it depends on local autopsy or neurosurgical case rates, logistical barriers in tissue procurements, and the willingness, communication, and coordination between neurosurgeons, pathologists, and research departments. Regulatory rules and laws differ internationally, further complicating data sharing and standardization. Technologically, maintaining tissue viability over time remains difficult, and ethical considerations such as robust informed consent procedures and donor privacy will always be tied closely to this kind of research.

Stem cell or iPSC-derived cells and organoids offer patient-specific and developmentally relevant platforms; however, they still lack key physiological features. Missing vasculature, absence of sensory input, and incomplete maturation of functional neuronal circuits limit their ability to model human brain states or functions. These model systems are rapidly evolving to meet these requirements and, as a consequence, have raised entirely new ethical questions (de Jongh et al., 2022; Kataoka et al., 2025). Concerns about rudimentary forms of consciousness, even though currently hypothetical, have led to intense discussions about ethical thresholds and the moral status of organoids (Boyd and Lipshitz, 2024). Also, the transplantation of organoids in animal hosts or brain slices (Panagiotakopoulou et al., 2025) could theoretically enhance the animal's or slice's cognitive capacities and thus, increase its moral status along with corresponding ethical considerations and regulatory restrictions (Chen et al., 2019). These complex developments would additionally impact the donor consent process, requiring much more explicit donor information, as well as clear legal definitions of tissue ownership, data rights, and patentability.

Moreover, inter-individual variability is a huge factor in human experimentation and differentiates it substantially from animal-based studies. Human variability originates from differences in donor age, sex, medical history, genetic background, lifestyle, and many other factors, and can make the interpretation of human data extremely difficult. While some researchers see such heterogeneity as a curse, as it challenges controlled experimental design, standardization, and complicates delineating the impact of all these factors on the experimental outcome, it could also potentially be seen as a blessing. After all, human heterogeneity accurately reflects the diversity of the population that ultimately will be the target audience of any translational preclinical research.

## 5 Conclusion

In order to overcome these limitations, continued innovation will be needed in all fields concerning human-based neuroscientific research, including bioengineering, technical standardization, data interpretation, consent procedures, donor autonomy and privacy, ethical oversight, and regulatory affairs. Nevertheless, working toward vascularized, interconnected, and physiologically relevant human model systems will tremendously increase the translational value in neuroscientific research and pave the way for their broader adoption as complementary or alternative platforms to animal experimentation. Ultimately, simple *in vitro* as well as animal experiments remain irreplaceable in neuroscientific research, but future approaches should incorporate a human-centered experimental pipeline aimed at cross-validating preclinical therapeutic candidates and therefore narrow down the translational gap toward actual clinical application in the human system.
